# Supplementary material for: Development of an adeno-associated virus vector for gene replacement therapy of NF1-related tumors
Source: Nat Commun. 2025 Sep 29;16:8594. doi: 10.1038/s41467-025-63619-4 (PMC12480499; doi:10.1038/s41467-025-63619-4)
Supplement: Supplementary file 1 — Supplementary Information [file 41467_2025_63619_MOESM1_ESM.pdf]

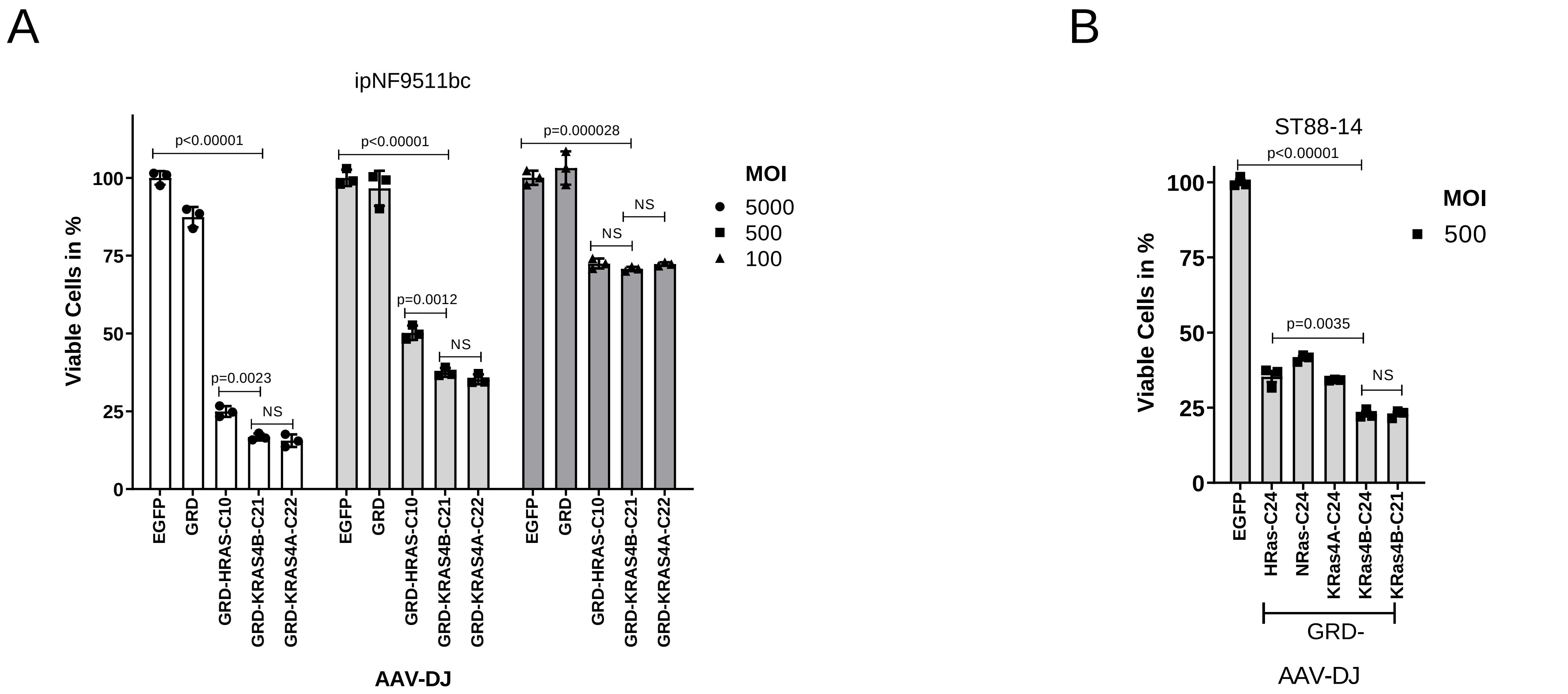

Supplementary Figure 1. Inhibition of NF1 cells by NF1-GRD fused to different HVR C-terminal sequences of RAS.

A. Inhibition of ipNF9511bc cells by GRD fused with various Ras hypervariable region (HVR) sequences. ipNF9511bc cells were transfected with indicated GRD constructs packaged in AAV-DJ at different multiplicity of infection (MOI). After 3 days, viable cells were measured via WST-8 at Abs 450 nm, with AAV-DJ-EGFP as control (100%). Three biological replicates were performed. Data are presented as mean values with SD and analyzed by t-test.

B. Inhibition of ST88-14 cells by GRD fused with various Ras HVR sequences. ST88-14 cells were transfected with indicated GRD constructs packaged in AAV-DJ at the MOI of 500. After 3 days, viable cells were measured via WST-8 at Abs 450nm, with AAV-DJ-EGFP as control (100%). Three biological replicates were performed. Data are presented as mean values with SD and analyzed by t-test.

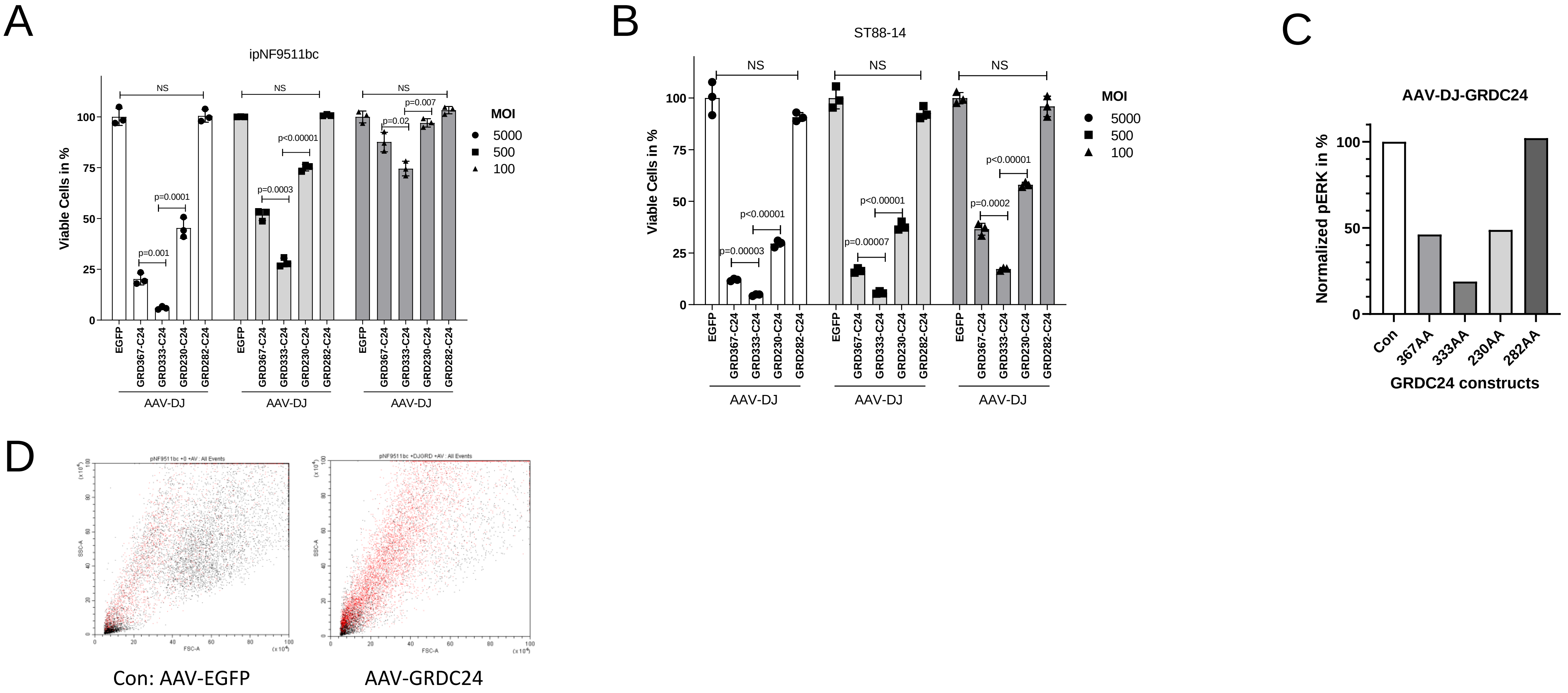

Supplementary Figure 2. Optimization of NF1 GRD sequences attached to 24 AA of KRAS4B C-terminus.

A and **B**. Inhibition of ipNF9511bc and ST88-14 cells by various length of GRD sequences fused with KRAS4B-C24 sequence. ipNF9511bc cells were transfected with indicated GRD constructs (GRD367: AA 1172-1538, GRD333: AA 1200-1532, GRD230: AA 1248-1477, GRD282: AA 1222-1503) packaged in AAV-DJ at different MOIs. After 3 days, viable cells were measured via WST-8 at Abs 450 nm, with AAV-DJ-EGFP as control (100%). Three biological replicates were performed. Data are presented as mean values with SD and analyzed by t-test.

C. Semi-quantification of normalized pERK1/2 signals in Fig. 1E. The total density of anti-pERK1/2 and anti-βActin was measured by ImageJ. Anti-pERK1/2 signals were normalized by the anti-βActin control.

D. SSC and FSC blots of flowcytometry in Figure 1H.

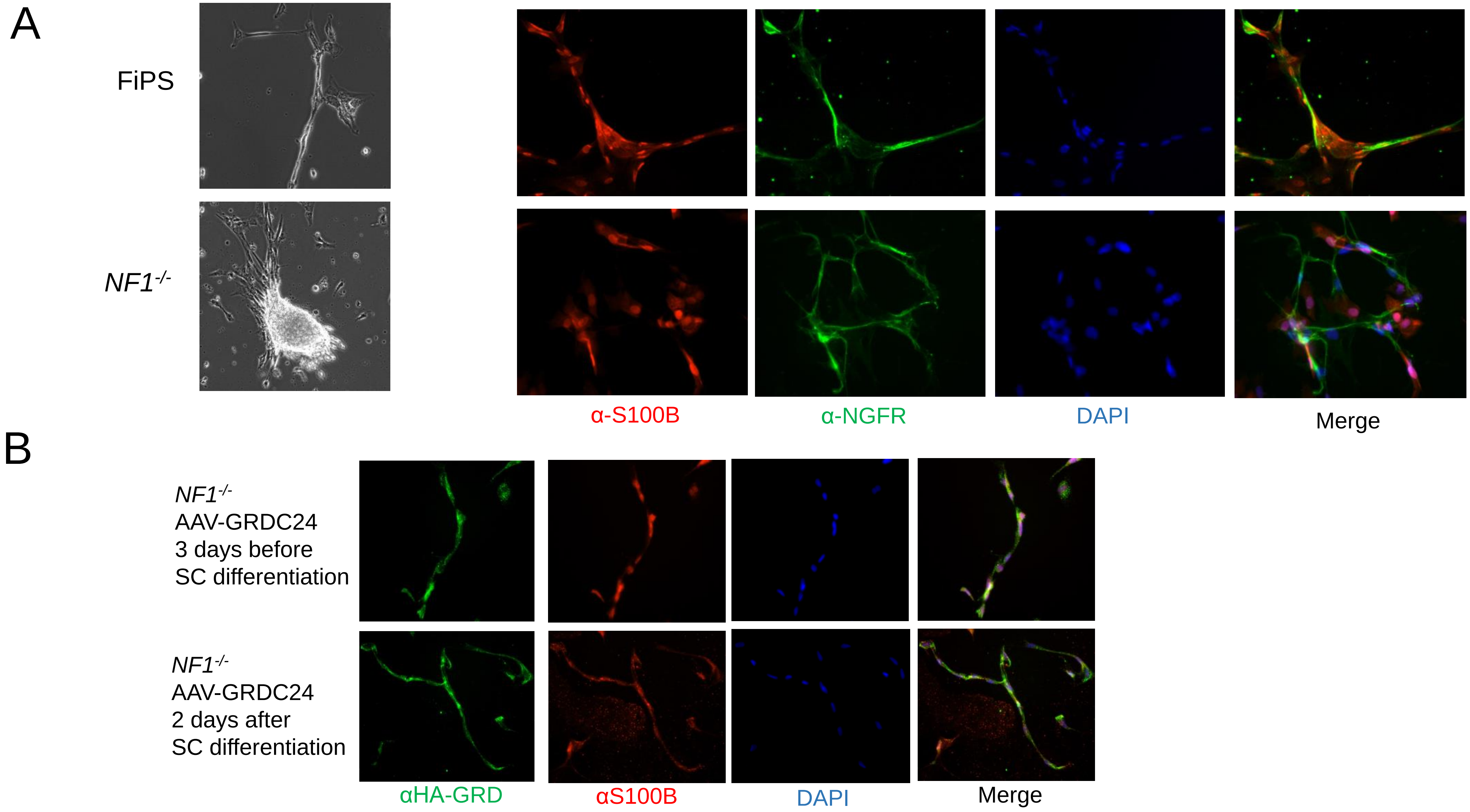

Supplementary Figure 3. Schwann cell differentiation of NF1 WT or NF1-/- iPSCs

A. Morphology of FiPS and NF1-/- cells after Schwann cell (SC) differentiation. FiPS or NF1-/- neural crest (NC) cells were incubated in the SC media for 7 days (left panel) or 20 days (right panel) and the cell morphology showed far less organized in NF1-/- population than that in FiPS (left panels).

B. FiPS and NF1-/- NCs transfected by AAV-DJ-GRDC24 at indicated time and were differentiated to SC in SC media for 20 days. The cell morphology showed similar features as FiPS cells in A, in contrast to that of NF1-/- cells.

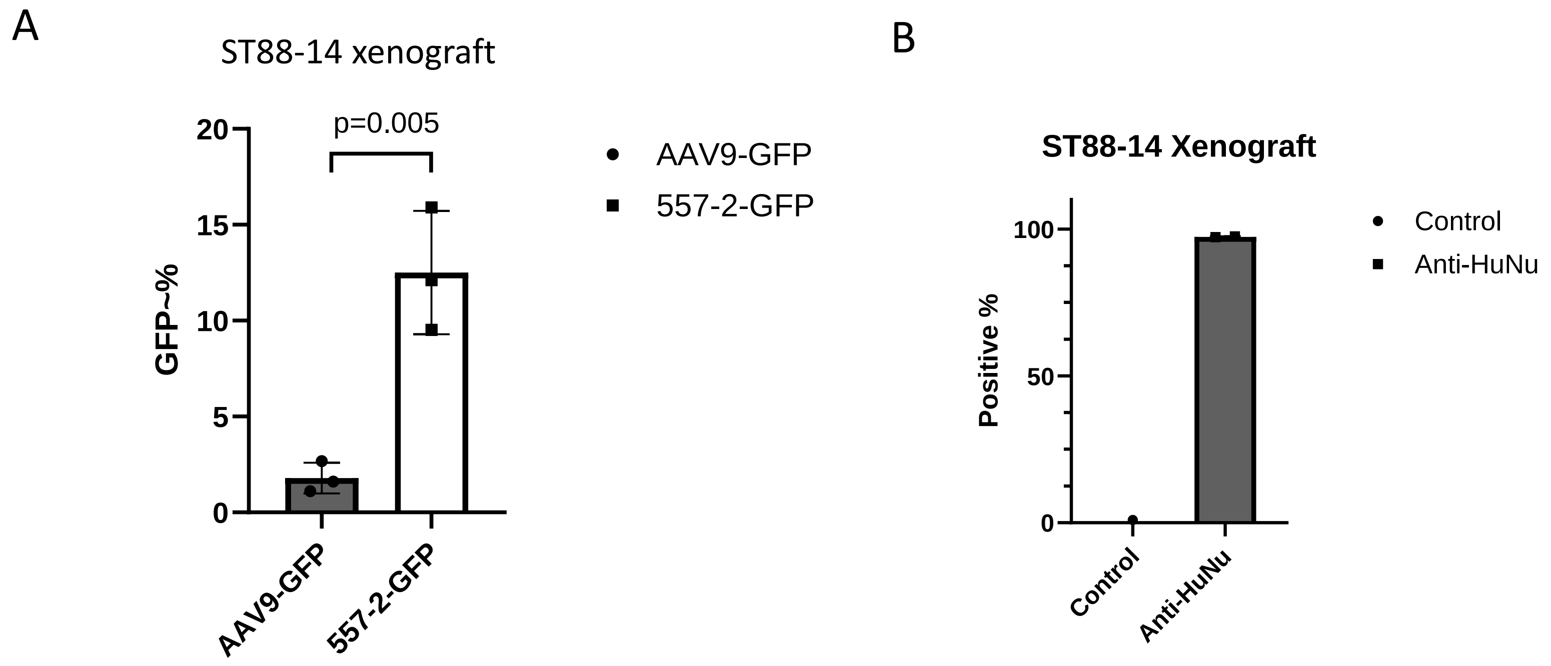

Supplementary Figure 4. Transduction of different AAVs

A. Rates of GFP transduction in ST88-14 xenograft tumors after AAV9-GFP or AAV-557-2-GFP IV injection. Tumor-bearing mice were injected with 1x1012 vg (viral genome) of the indicated AAV vector and after 14 days, tumors were harvested and dissociated, and GFP-positive cells were quantified using flowcytometry (n=3 tumors). Data are presented as mean values with SD and analyzed by two-tailed t-test.

B. Human nuclear antigen staining of ST88-14 tumor confirmed the human origin of the tumor cells. ST88-14 xenograft tumor was dissociated and stained with anti-human nuclear antigen antibody. The control was stained with only the secondary antibody. Three biological replicates were performed.

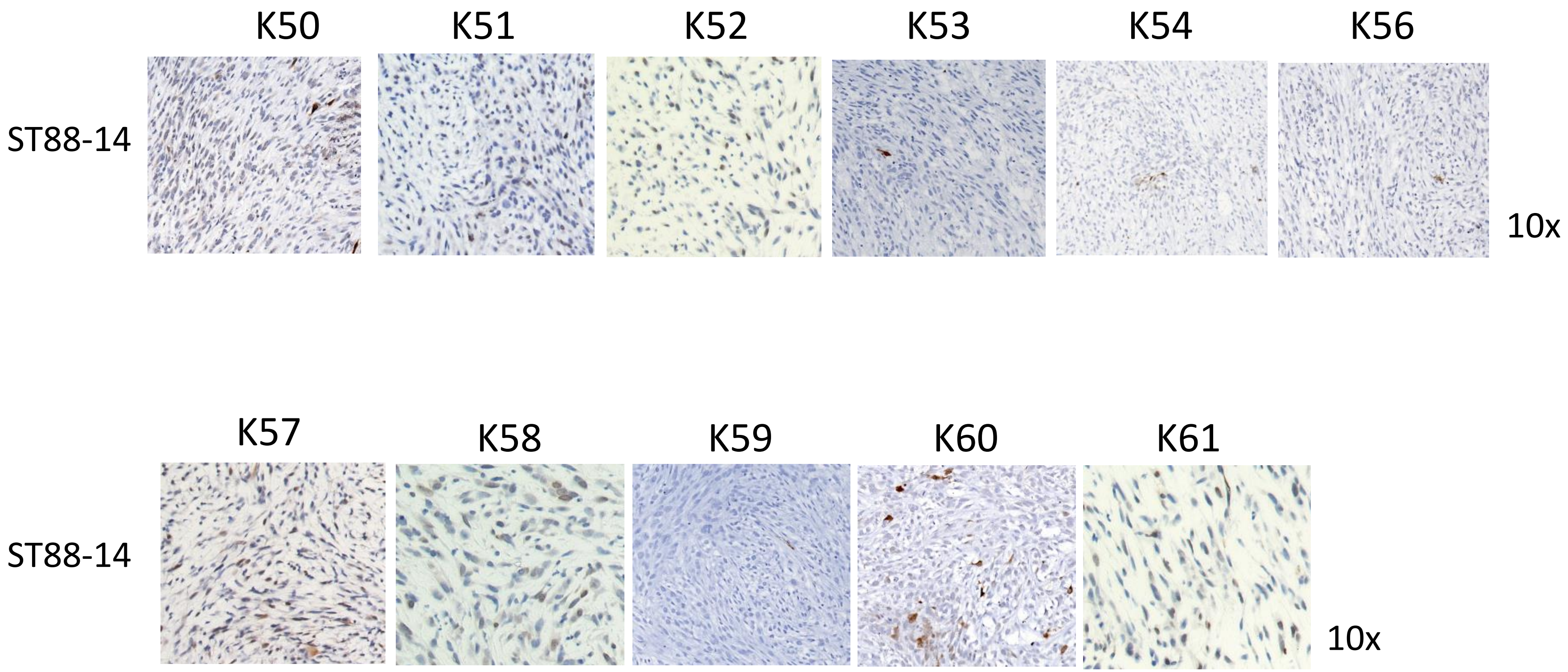

Supplementary Figure 5. Comparison of GFP transduction by different AAV mutants.
The top capsid candidates (not including K55) from the selection of random peptide library (Fig. 4B) were used to package GFP and IV injected in ST88-14-bearing NSG mice. Anti-GFP IHC of the tumors was performed.

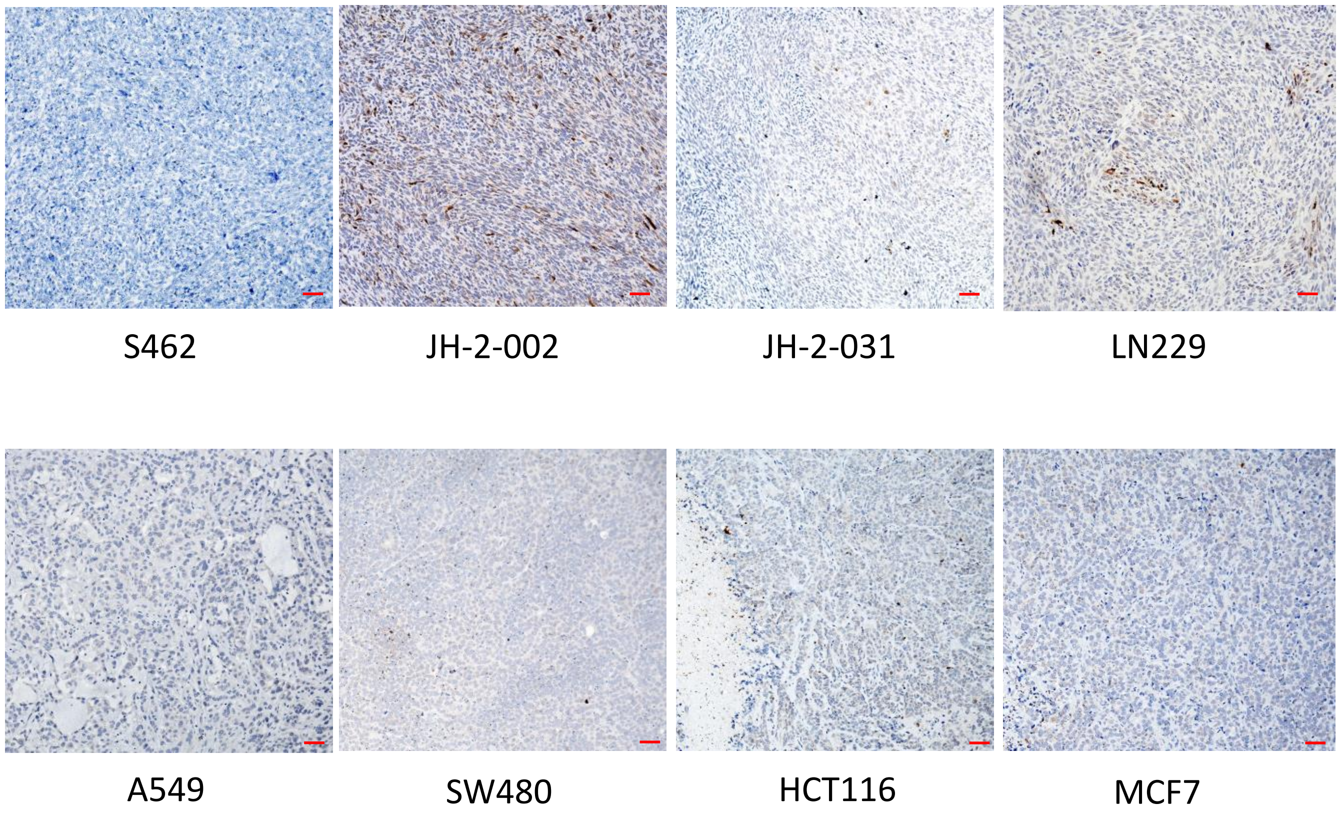

Supplementary Figure 6. IHC of xenograft tumors transduced by AAV-K55-GFP

Distribution of AAV-K55 in NF1 xenografts and PDXs, including the subcutaneous LN229 NF1-/- glioma, and a panel of solid human xenograft tumors. AAV-K55-GFP was injected IV at the dose of 10^{12} vg and tumors were harvested and anti-GFP IHC was performed using DAB as visualization and hematoxylin as counterstain. Scale bar = 50 µm.

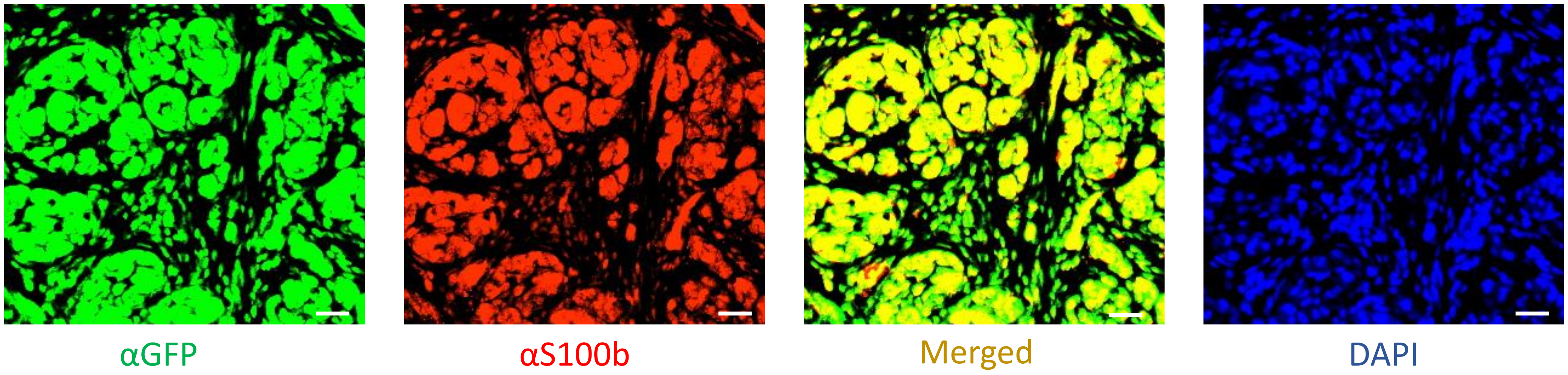

Supplementary Figure 7. Higher magnification of the GFP- and S100B-stained sections in Figure 5B.
AAV-K55-GFP transduced mostly the S100B-positive population (tumor) in 3MM-FB neurofibroma xenograft. A section was stained with anti-GFP (green) and anti-S100B (red) antibodies. Yellow color in the merged picture indicates the co-localization of anti-GFP (green) and anti-S100B (red) staining. Scale bar = 25 µm.

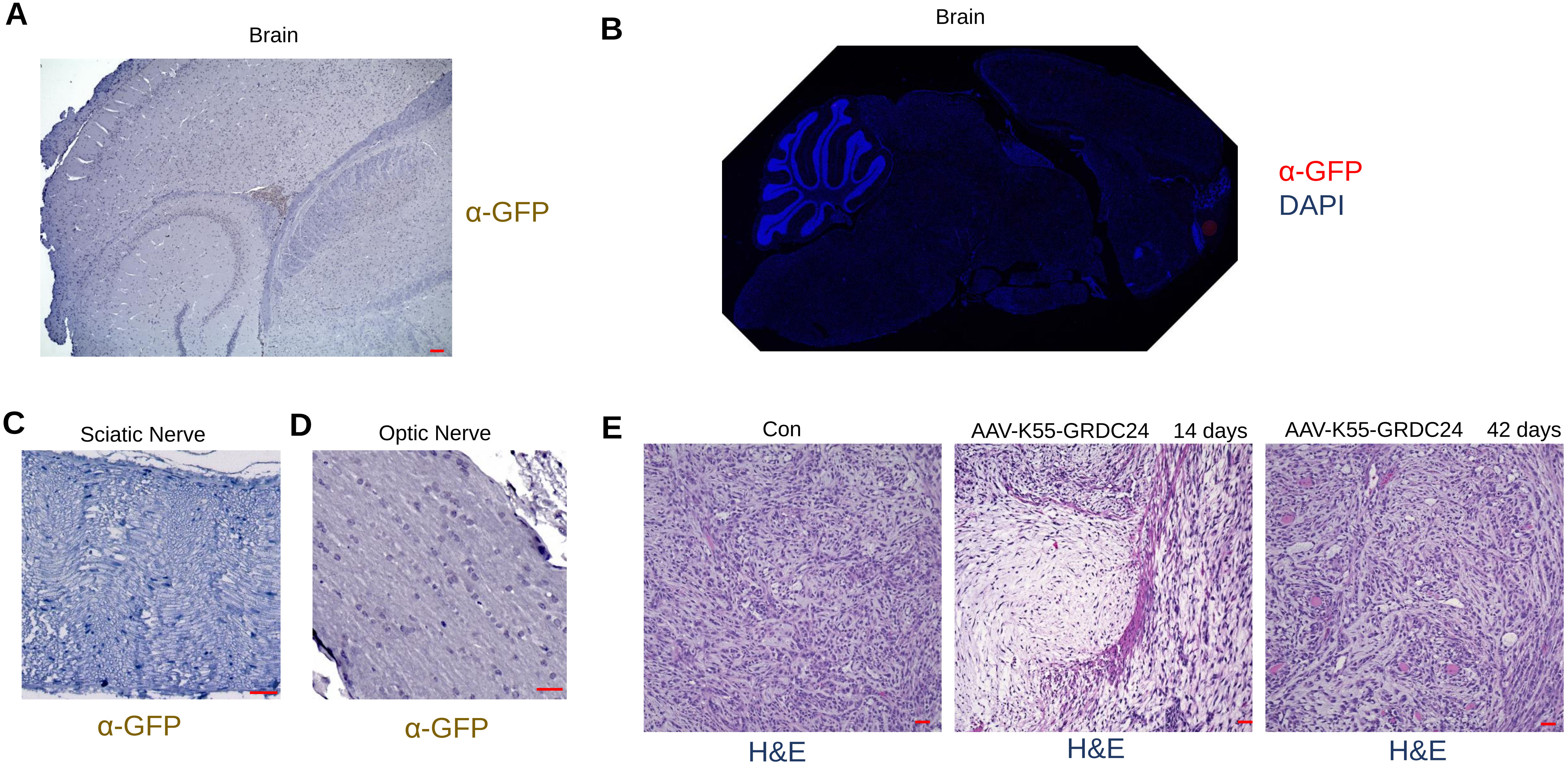

Supplementary Figure 8. Limited distribution of AAV-K55 in the brain and nerves.
AAV-K55-GFP was injected IV in NSG mice and the brain, sciatic nerve and optic nerve were harvested after 14 days. Anti-GFP immunohistochemistry indicated limited GFP expression in the brain (**A** and **B**, scale bar = 100 µm) and no detectable expression in the sciatic and optic nerve (**C** and **D**, scale bar = 20µm). In **E**, ST88-14 xenograft tumor-bearing mice were treated with AAV-K55-GRDC24 IV at 10^{12} vg and the tumor sections of untreated control, day 14 and day 42 after the treatment were stained by H&E (scale bar = 50 µm).

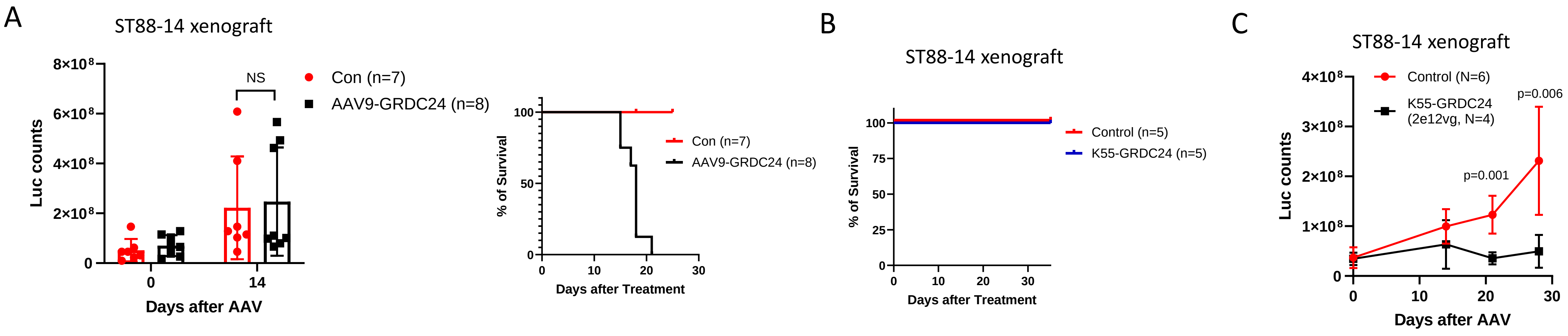

Supplementary Figure 9. Treatment with AAV9-GRDC24 and a higher dose of AAV-K55-GRDC24

A. AAV9-GRDC24 show no treatment efficacy in ST88-14 xenograft tumors. NSG mice were implanted with ST88-14-luc cells and after 14 days, were injected IV with AAV9-GRDC24 at 10^{12} vg (n = 8 mice). Controls were untreated animals (n = 7 mice). Tumor sizes were evaluated before and 14 days after the treatment and animals were also treated with 5 mg/ml dexamethasone IP shortly before the AAV injection and 3 times a week after. All treated mice died between day 15 and 21. Data are presented as mean values with SD and analyzed by one-tailed t-test.

B. Survival of ST88-14-luc xenograft-bearing mice treated with AAV-K55-GRDC24 at 1012 vg as shown in Fig. 6A. No animal died during the treatment course (n=5 mice)

C. Treatment with 2×10^{12} vg of AAV-K55-GRDC24 significantly suppressed the growth of ST88-14 xenograft tumors. NSG mice were implanted with ST88-14-luc cells in the sciatic nerve and after 14 days, were injected IV with AAV-K55-GRDC24 at 2×10^{12} vg (n = 4 mice). Controls were untreated animals (n = 5 mice). Tumor sizes were evaluated before and after the treatment using IVIS imaging. Animals were treated with 5 mg/ml dexamethasone IP shortly before the AAV injection and 3 times a week after. Data are presented as mean values with SD and analyzed by one-tailed t-test.

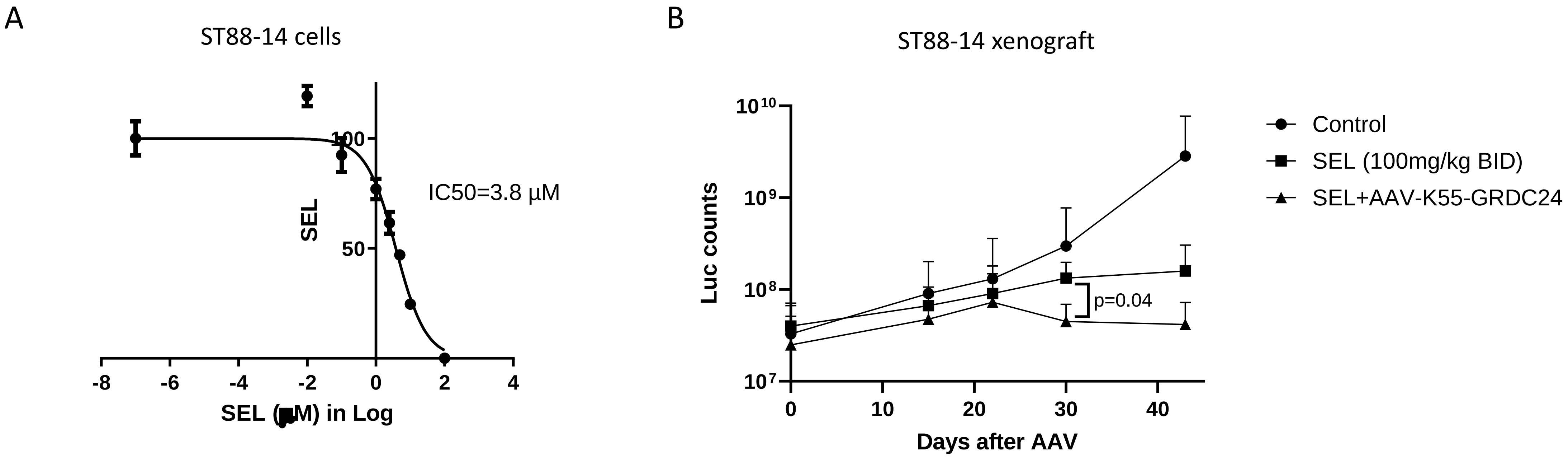

Supplementary Figure 10. Treating ST88-14 xenograft tumors with selumetinib (SEL) in combination of AAV-K55-GRDC24
A. IC50 of ST88-14 cells with selumetinib was determined at 3.8 µM. ST88-14 cells were incubated with a series of selumetinib dilutions for 72 hr and the viable cells were measured by Cell Counting Kit-8 (CCK-8). Three biological replicates were performed.
B. ST88-14-luc cells were implanted in the sciatic nerve of NSG mice and after 14 days, the animals were treated with selumetinib at 100 mg/kg twice daily via oral gavage with or without IV injection of 10^{12} vg AAV-K55-GRDC24 (n=4 mice). Data are analyzed by two-tailed t-test.
