# Supplementary material for: Development of an adeno-associated virus vector for gene replacement therapy of NF1-related tumors
Source: Nat Commun. 2025 Sep 29;16:8594. doi: 10.1038/s41467-025-63619-4 (PMC12480499; doi:10.1038/s41467-025-63619-4)
Supplement: Supplementary file 2 — Description of Additional Supplementary Files [file 41467_2025_63619_MOESM2_ESM.pdf]

Description of Additional Supplementary Files

Supplementary Data 1. DNA and amino acid sequences of the AAV capsids created in DNA shuffling and random peptide library screening.

Supplementary Data 2. Selected candidates from random peptide library screening

Supplementary Data 3. NSG data of random peptide library selection
